# Csf1r or Mer inhibition delays liver regeneration via suppression of Kupffer cells

**DOI:** 10.1371/journal.pone.0216275

**Published:** 2019-05-01

**Authors:** Juan A. Santamaria-Barria, Shan Zeng, Jonathan B. Greer, Michael J. Beckman, Adrian M. Seifert, Noah A. Cohen, Jennifer Q. Zhang, Megan H. Crawley, Benjamin L. Green, Jennifer K. Loo, Joanna H. Maltbaek, Ronald P. DeMatteo

**Affiliations:** Department of Surgery, Memorial Sloan Kettering Cancer Center, New York, NY, United States of America; Montana State University Bozeman, UNITED STATES

## Abstract

**Introduction:**

Murine Kupffer cells (KCs) comprise CD11b^hi^ and F4/80^hi^ subsets. Tissue-resident macrophages are known to express the tyrosine kinase receptors colony-stimulating factor 1 receptor (Csf1r) and Mer. However, the expression of Csf1r and Mer on KC subsets and the importance of these tyrosine kinases during liver regeneration (LR) are unknown.

**Methods:**

KCs from wild-type and Csf1r-GFP mice were characterized by flow cytometry. Partial hepatectomy (PH) was performed in mice treated with clodronate liposomes, a Csf1r small molecule inhibitor or depleting antibody, or a small molecule Mer inhibitor. Sera and livers were analyzed. The function of sorted KC subsets was tested in vitro.

**Results:**

Mer was specifically expressed on tissue-resident F4/80^hi^ KCs, 55% of which also expressed Csf1r. Mer^+^Csf1r^+^ and Mer^+^Csf1r^-^ KCs had distinct expression of macrophage markers. Csf1r inhibition in mice reduced F4/80^hi^ KCs by approximately 50%, but did not affect CD11b^hi^ KCs. Clodronate liposomes depleted F4/80^hi^ KCs, but also altered levels of other intrahepatic leukocytes. Csf1r inhibition delayed LR, as demonstrated by a 20% reduction in liver-to-body weight ratios 7 days after PH. At 36h after PH, Csf1r inhibition increased serum ALT and histological liver injury, and decreased liver cell proliferation. A small molecule inhibitor of Mer did not alter the percentage of KCs or their proliferation and just modestly delayed LR. In vitro, Csf1r or Mer inhibition did not decrease KC viability, but did attenuate their cytokine response to stimulation.

**Conclusions:**

F4/80^hi^ KCs are Mer^+^ and can be subdivided based on Csf1r expression. Csf1r or Mer inhibition each reduces KC cytokine production and delays LR.

## Introduction

Macrophages residing in the liver are known as Kupffer cells (KCs) and represent the largest macrophage population in the body. KCs are important homeostatic regulators of the liver’s immune tolerogenic immune environment and mediators of liver injury and repair.[1] Several phenotypic and functional KC subsets have been identified.[2–5] CD11b^hi^ KCs are small, radiosensitive, cytokine-producing cells that are dispersed throughout the liver parenchyma. In contrast, F4/80^hi^ KCs are large, radioresistant cells that are located in the liver sinusoids and have potent phagocytic function.[3, 5] In a model of orthotopic liver transplantation in bone marrow-chimeric mice, KCs have also been distinguished as either long-lived, tissue-resident macrophages of the liver or derived from monocytic precursors originating in the bone marrow.[6]

Liver regeneration (LR) is the only biologic process in mammals in which organ volume and function completely reconstitute after significant tissue loss.[7] LR has clinical significance in liver trauma and transplantation. Depletion studies employing dichloromethylene-biphosphonate (clodronate) liposomes have demonstrated a crucial role for KCs in LR.[8–10] However, because liposomes affect all KCs, and not just F4/80^hi^ KCs, the function of individual KC subsets is unclear.

Colony-stimulating factor 1 receptor (Csf1r) is a transmembrane tyrosine kinase receptor and the primary homeostatic regulator of the mononuclear phagocytic system, which includes KCs.[11] Csf1r and its ligand, monocyte colony-stimulating factor 1 (M-CSF; CSF1), regulate macrophage proliferation, function, survival, and migration.[11, 12] Therapies involving Csf1r and M-CSF are currently under investigation in clinical trials for cancer, inflammatory diseases, and tissue repair.[11] The effects of inhibiting or stimulating Csf1r in normal animals during LR are unknown.

Mer (Mertk) is a member of the TAM receptor tyrosine kinase family, which also includes Tyro-3 and Axl. In macrophages, Mer controls the phagocytosis of apoptotic cells and negatively regulates the innate immune response.[13–15] A large-scale gene expression study of murine macrophages found that Mer and CD64 were the most specific markers of tissue-resident macrophages.[16] However, the analysis was based only on macrophages from the peritoneum, splenic red pulp, lung, and brain. KCs were not analyzed due to their variable definitions and inability to be purified. The expression of Mer by KCs and its role in LR are unknown. Herein, we sought to determine the importance of Csf1r and Mer in LR by using a mouse model of partial hepatectomy (PH).

## Materials and methods

### Animals and reagents

Eight- to 10-week old male mice were purchased from the Jackson Laboratory (Bar Harbor, ME); the strains were C57BL/6J (B6), B6-Tg(Csf1r-EGFP-NGFR/FKBP1A/TNFRSF6)2Bck/J (Csf1r-GFP mice),[17] B6;129-Mertk^tm1Grl^/J (Mer^-/-^ mice),[18] and B6129SF2/J (Mer^+/+^ wild-type control mice, purchased separately and cohoused). PLX5622, a small molecule Csf1r tyrosine kinase inhibitor,[19] was provided by Plexxikon (Berkeley, CA) and administered as a formulated diet chow (1200 mg/kg). Rat anti-mouse Csf1r (clone AFS98) monoclonal antibody or rat IgG2a isotype control (clone 2A3), both from BioXCell (West Lebanon, NH), was administered at a loading dose of 500 μg i.p. on day 1 followed by 250 μg on days 3 and 5. Clodronate liposomes and PBS liposomes (clodronateliposomes.org, Netherlands) were administered by tail vein injection at a dose of 50 mg/kg. UNC2250, a selective Mer inhibitor,[20] was purchased from Selleckchem (Houston, TX) and administered by oral gavage at a dose of 3 mg/kg twice daily. Mice were housed and bred in a pathogen-free facility at Memorial Sloan Kettering Cancer Center. The Institutional Animal Care and Use Committee of the Memorial Sloan Kettering Cancer Center approved all procedures. All animals received humane care according to the criteria outlined in the “Guide for the Care and Use of Laboratory Animals” prepared by the National Academy of Sciences and published by the National Institutes of Health.

### Partial hepatectomy model

Two-thirds PH was performed as previously described, with minor modifications.[21] Briefly, mice were anesthetized with inhaled isoflurane and placed supine. An upper midline abdominal incision was made to expose the liver. The vascular pedicles of the left and median liver lobes were ligated and divided. The abdominal incision was closed and 1 ml of sterile saline was injected subcutaneously. Sham surgery was performed without removing the liver lobes. All surgeries were done without a fasting period and during the light cycle, and mice had access to food and water at all times. Mice were euthanized with carbon dioxide, liver-to-body weight ratios were calculated, and blood was collected via cardiac puncture. Sera were analyzed for alanine aminotransferase (ALT) using the Olympus AU 400 Chemistry Analyzer (Laboratory of Comparative Pathology, Sloan Kettering Institute). Serum cytokines were measured using a cytometric bead array according to the manufacturer’s instructions (Mouse Inflammation kit, BD Biosciences). Livers were immediately fixed in 4% paraformaldehyde, embedded in paraffin, and cut to 5-μm-thick sections. Tissues were stained with hematoxylin and eosin and Oil Red O. Immunohistochemistry for proliferating cell nuclear antigen (PCNA) was performed with the PCNA kit from Dako (Carpinteria, CA). Slides were assessed for inflammation, tissue damage, and PCNA^+^ cells using an Axio Imager 2 wide-field microscope (Zeiss, Germany).

### Isolation of liver nonparenchymal cells (NPCs)

Liver NPCs were isolated as previously described,[22] with modifications. After the liver was harvested and processed, the supernatant was centrifuged (450 *g* for 5 minutes) to isolate NPCs, which were further enriched by a 40% Optiprep (Sigma-Aldrich) density gradient per the manufacturer’s instructions. The layer of low density cells at the interface was harvested.

### Flow cytometry

Flow cytometry was performed on a FACSAria (BD Biosciences, San Jose, CA). Fc receptors were blocked with 1 μg anti-FcγRIII/II antibody (2.4G2; BioXCell) per 10^6^ cells. Cells were stained with fluorescent-conjugated antibodies against CD11b (APC-Cy7; clone M1/70), CD19 (APC-Cy7; clone 1D3), NK1.1 (PE, APC-Cy7; clone PK136), CD45 (FITC; clone 30-F11), CD19 (PE-Cy7; clone SJ25C1), CD8 (AF 700; clone 53–6.7), CD4 (APC-Cy7; clone RM4-5), CD64 (PE; clone X54-5/7.1), CD16/32 (PE; clone 2.4G2), Ly6c (PerCP/cy5.5; clone AC-21), Flt-3 (PE; clone A2F10.1), CD80 (PE; clone 16-10A1), CD86 (PE-cy7; clone GL1), Siglec-F (PE; clone E50-2440), CD14 (APC-Cy7; clone rmC5-3), and Kit (CD117; PerCP/Cy5.5; clone 2B8), all from BD Biosciences; Siglec-H (FITC; clone 551), CD45 (PerCP/Cy5.5; clone 30-F11), Ly6g (AF 700; clone 1A8), CD11c (PE/cy7; clone N418), MHC-II (AF 700; clone M5/114.15.2), CD3 (PerCP/Cy5.5; clone 17A2), CD68 (FITC; clone FA-11), and CX3CR1 (PE; clone SA011F11), all from BioLegend (San Diego, CA); and Axl (PE; clone 175128), Mer (APC; clone 108928), Tyro-3 (PE; clone 109646), and CCR2 (APC; clone 475301), all from R&D Systems (Minneapolis, MN); and F4/80 (APC; clone BM8) from Invitrogen (Carlsbad, CA). Isotype controls were used when applicable. For intracellular staining, we used the Fixation and Permeabilization Buffer Kit from eBioscience (Santa Clara, CA). Data were analyzed using FlowJo software (Tree Star, Ashland, OR). Histograms are shown with staining intensity on the *x*-axis and percentage of maximum on the *y*-axis.

### Immunofluorescence

Liver specimens were fixed in 4% paraformaldehyde, embedded in paraffin, and sectioned to 5 μm thickness. Antigen retrieval was achieved with citrate buffer. Tissue sections were stained overnight at 4°C with anti–mouse F4/80 (1:100 dilution; clone BM8), Csf1r (1:200 dilution; clone AFS98; both BioLegend), or Ki-67 (1:200 dilution; clone 16A8; Vector Laboratories). After being washed with PBS, slides were incubated with a fluorochrome-conjugated secondary antibody and then analyzed and imaged on an Axio Imager 2 wide-field microscope (Zeiss).

### Western blot

Whole protein was extracted from frozen liver tissue using ice-cold lysis buffer (10 mM Tris HCl, pH 7.5, 150 mM NaCl, 2 mM EDTA, 1% Triton X-100, 10% glycerol, 2 mM sodium orthovanadate), and cOmplete protease inhibitor cocktail (Roche Diagnostics, Indianapolis, IN). Protein concentrations were quantified using the Bradford method. Equal amounts of protein were separated by sodium dodecyl sulfate-polyacrylamide gel electrophoresis and transferred to a nitrocellulose membrane. The membrane was then probed with antibodies against phospho-Mer (ab192649, 1:500 dilution; Abcam, Cambridge, UK), total Mer (sc-365499, 1:200 dilution; Santa Cruz Biotechnology, Dallas, TX), phospho-STAT3 (Ser727; Y705, 1:1000 dilution; Cell Signaling Technology, Danvers, MA), and GAPDH (D16H11; Cell Signaling Technology).

### In vitro assays of cytokine production and viability

Using a FACSAria (BD Biosciences), CD45^+^Ly6c^-^Ly6g^-^Siglec-F^-^ NPCs from Csf1r-GFP mice were sorted into three KC subsets to >90% purity: CD11b^hi^, CD11b^int^ Mer^+^Csf1r^-^, and CD11b^int^Mer^+^Csf1r^+^. CD11b^int^Mer^+^ KCs from treated wild-type mice were sorted in a similar fashion. A viability dye was utilized. Cells were seeded in triplicates in 200 μL medium in flat-bottom 96-well plates and treated overnight with 2.5 μM PLX5622 or 2.5 μM UNC2250. The next day, 250 ng/mL LPS was added and 4h later supernatant was collected and cytokines were measured using a cytometric bead array (Mouse Inflammation kit, BD Biosciences), per the manufacturer’s instructions. For viability assays, cells were plated in flat-bottom 96-well plates and treated overnight with 2.5 or 5 μM PLX5622 or 100 ng/mL mouse recombinant M-CSF (BioLegend). Viability was determined according to the manufacturer’s instructions (Dojindo, Japan) and assessed by optical density at 450 nm.

### Quantitative RT-PCR

NPCs were obtained from wild-type mouse livers. After we blocked Fc receptors, F4/80^hi^ KCs were positively selected using F4/80 MicroBeads from Miltenyi (Germany), following the manufacturer’s instructions. Cells were plated in flat 6-well plates in medium and treated overnight with 2.5 or 5 μM PLX5622. The next day, 250 ng/mL LPS was added for 4h and RNA was isolated from the cells using the RNeasy Mini Kit by Qiagen (Valencia, CA), according to the manufacturer’s instructions. For cDNA synthesis, 0.5 μg of total RNA was transcribed with the TaqMan Reverse Transcription Reagents Kit using random hexamers (Applied Biosystems, Waltham, MA). PCR TaqMan probes for murine IL-6, TNF-α, and GAPDH from Applied Biosystems were used. Quantitative RT-PCR was performed using the ABI 7900 system (Applied Biosystems) and relative mRNA expression levels were calculated by the 2^-ΔΔCt^ method.

### Statistics

Results are expressed as mean +/- standard error of the mean (SEM). Statistical significance was determined by two-tailed Student’s t test or ANOVA as appropriate, using statistical software (Prism; GraphPad Software, La Jolla, CA). A *p value of* <0.05 was considered significant.

## Results

### Csf1r and Mer define mouse KC subsets

**Fig 1:**
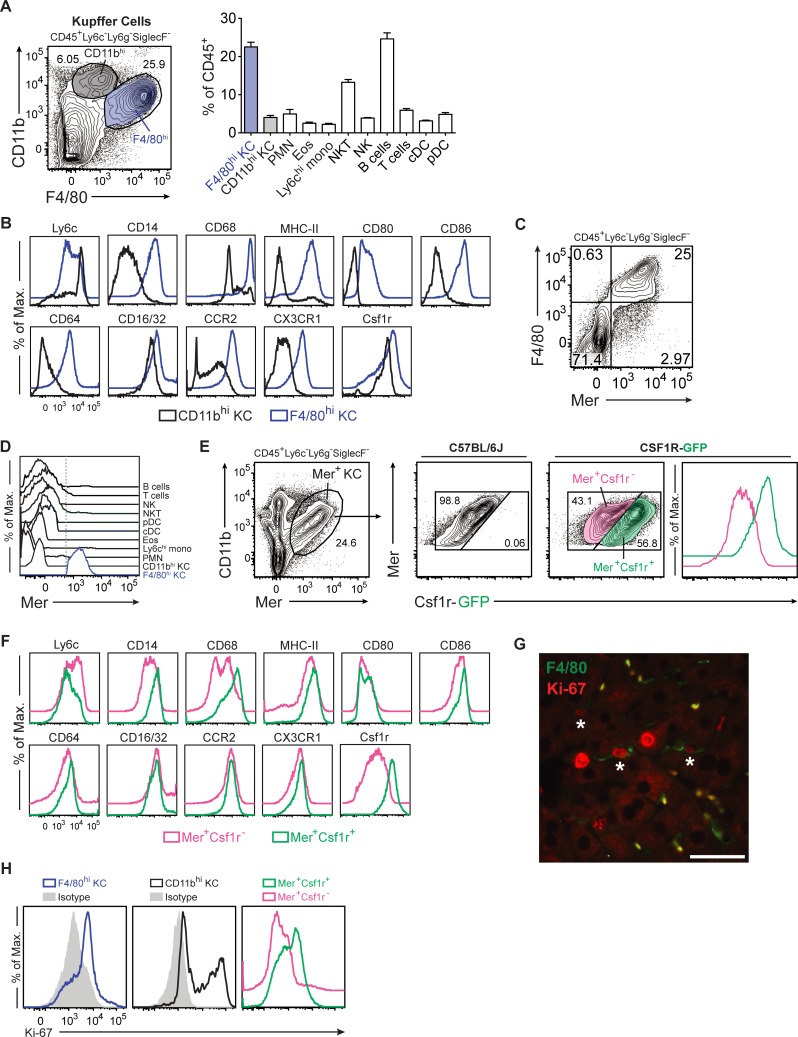
Csf1r and Mer expression further define KC subsets in mouse liver. **(A)** F4/80^hi^ KCs (blue, 23% of NPCs on average) and CD11b^hi^ KCs (gray, 4% of NPCs) are shown after excluding dendritic cells, monocytes, neutrophils and eosinophils from liver NPCs. PMN, neutrophils; Eos, eosinophils; Ly6c^hi^ mono, inflammatory monocytes; NKT, natural killer T cells; NK, natural killer cells; cDC, conventional dendritic cells; pDC, plasmacytoid dendritic cells. **(B)** Expression of various markers on F4/80^hi^ (blue) and CD11b^hi^ (gray) KCs by flow cytometry. Csf1r expression was determined in Csf1r-GFP mice. **(C)** F4/80 and Mer expression on KCs by flow cytometry. **(D)** Mer expression on liver NPCs by flow cytometry. (**E)** GFP expression in Mer^+^ KCs from Csf1r-GFP mice compared to C57BL/6J mice, which do not express GFP, showing Mer^+^Csf1r^-^ KCs (pink, 43% of Mer^+^ KCs) and Mer^+^Csf1r^+^ KCs (green, 57% of Mer^+^ KCs). **(F)** Expression of various markers on Mer^+^Csf1r^-^ (pink) and Mer^+^Csf1r^+^ (green) KCs. **(G)** Photomicrograph of mouse liver demonstrating co-localization of F4/80 (green) and nuclear Ki-67 (red) staining in KCs (white asterisks) from untreated mice by immunofluorescence. Scale bar, 50 μm. **(H)** Intracellular Ki-67 expression in F4/80^hi^ (blue histogram, left), CD11b^hi^ (black histogram, middle), Mer^+^Csf1r^-^ (pink histogram, right) and Mer^+^Csf1r^+^ (green histogram, right) KCs. Data represent at least 2 independent experiments with 3 to 5 mice per group.

Using a traditional flow cytometry gating strategy,[3] we identified CD11b^hi^ and F4/80^hi^ KCs from untreated mice (**Fig 1A and S1A Fig**). The two KC subsets had distinct expression of various markers (**Fig 1B**). Notably, F4/80^hi^ KCs had higher expression of Ly6c, but less than Ly6c^hi^ inflammatory monocytes, which had been excluded by our gating strategy. F4/80^hi^ KCs also expressed higher amounts of CD14 and CD68, co-stimulatory molecules, Fc receptors, chemokine receptors, and Csf1r.

Previous studies have described tissue-resident and bone marrow-derived macrophages as ontogenetically distinct entities in various tissue compartments.[6, 23, 24] Mer was identified as one of the most specific markers for tissue-resident macrophages outside the liver.[16] Within the liver, we found that Mer was expressed by all F4/80^hi^ KCs, but not by F4/80^-^ (i.e., CD11b^hi^) KCs or other liver NPCs (**Fig 1C and 1D, and S1A Fig**). In contrast, the related tyrosine kinases Tyro-3 and Axl were less specific for F4/80^hi^ KCs (**S1B Fig**). Importantly, hepatocytes and liver sinusoidal cells do not express Mer.[25]

Csf1r regulates survival and function of mononuclear phagocytes.[11, 12] Previous data showed that Csf1r is predominantly expressed in tissue-resident macrophages.[26] We found that Csf1r frequently co-localized with F4/80^+^ cells in the liver by immunofluorescence (**S1C Fig**). To further define Csf1r expression on Mer^+^ KCs, we utilized transgenic Csf1r-GFP mice and found that 57% of Mer^+^ KCs expressed GFP (**Fig 1E**). Thus, F4/80^hi^ KCs contained distinct Mer^+^Csf1r^+^ and Mer^+^Csf1r^-^ subsets. Analysis of these subsets indicated that they had distinct phenotypic markers (**Fig 1F**) and different expression of other TAM family receptors (**S1D Fig**). Mer^+^Csf1r^+^ KCs expressed greater levels of CD14, CD68, co-stimulatory molecules, and CD64. Others have shown that tissue-resident macrophages are derived locally and do not originate from circulating bone marrow progenitors.[27, 28] Accordingly, F4/80^hi^ KCs stained for the proliferation marker Ki-67 by immunofluorescence (**Fig 1G**). Flow cytometry revealed more Ki-67 in Mer^+^Csf1r^+^ KCs than in Mer^+^Csf1r^-^ KCs, while CD11b^hi^ KCs had bimodal expression (**Fig 1H**). Collectively, these data demonstrate that F4/80^hi^ KCs selectively express Mer and comprise 2 subsets based on Csf1r expression.

### Csf1r inhibition, clodronate liposomes, and Mer inhibition have distinct effects

**Fig 2:**
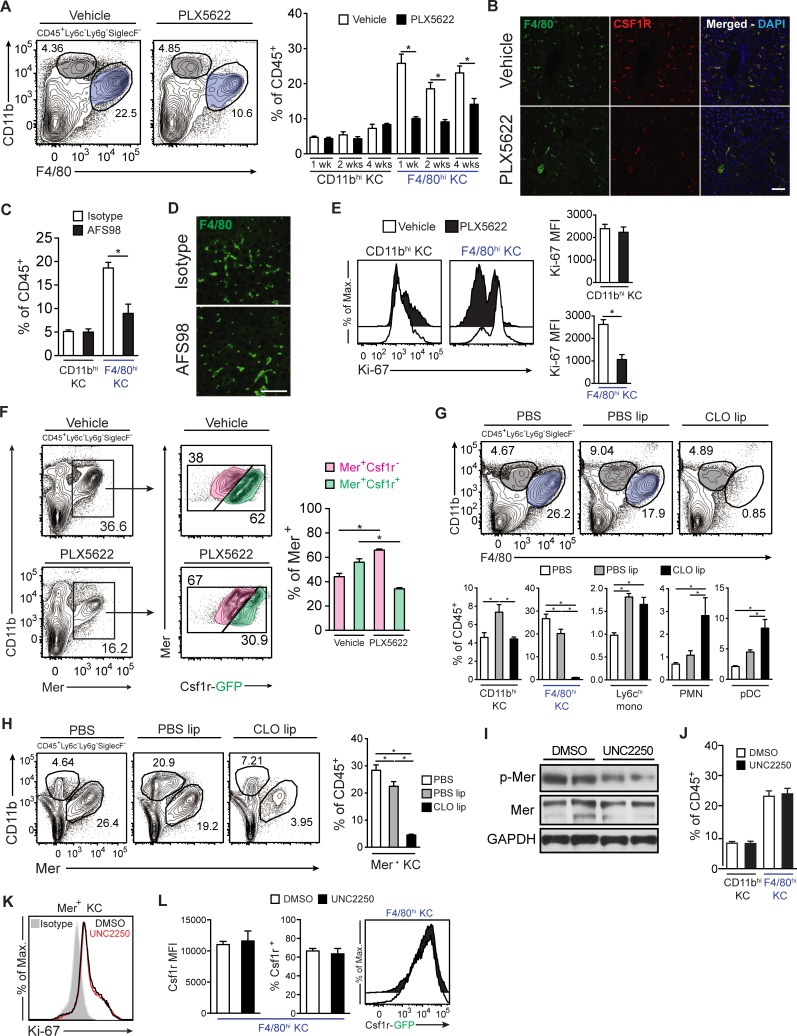
Csf1r inhibition, Mer inhibition, and clodronate liposomes have distinct biologic effects. **(A)** Representative flow cytometry of F4/80^hi^ (blue) and CD11b^hi^ (gray) KCs after 2 weeks of PLX5622 treatment (left). KC subsets after 1, 2, and 4 weeks of PLX5622 treatment (right). **(B)** Immunofluorescence photomicrographs of mouse livers after 2 weeks of PLX5622 treatment. Green indicates F4/80, red indicates Csf1r, and blue indicates the nuclear stain DAPI. Scale bar, 100 μm. **(C)** KC subsets and **(D)** immunofluorescence photomicrographs of mouse liver after AFS98 administration for 1 week. Green indicates F4/80. Scale bars, 50 μm. **(E)** Intracellular Ki-67 in F4/80^hi^ and CD11b^hi^ KCs after 1 week of PLX5622 treatment by flow cytometry (left) and median fluorescence intensity (MFI) (right). **(F)** Flow cytometry analysis (left) and quantification (right) of Mer^+^Csf1r^-^ (pink) and Mer^+^Csf1r^+^ (green) KCs after 2 weeks of PLX5622. **(G)** Flow cytometry analysis (top) and quantification (bottom) of F4/80^hi^ (blue) and CD11b^hi^ (gray) KCs, inflammatory monocytes (Ly6c^hi^ mono), neutrophils (PMN), and plasmacytoid dendritic cells (pDC) 24h after administration of clodronate and PBS liposomes. **(H)** Flow cytometry analysis (left) and quantification (right) of Mer^+^ KCs 24h after administration of clodronate and PBS liposomes. **(I)** Protein expression of phosphor-Mer and total Mer by Western blot from whole-liver lysates after 1 week of treatment with UNC2250. **(J)** Quantification of F4/80^hi^ and CD11b^hi^ KCs after 1 week of UNC2250. **(K)** Flow cytometry histogram analysis of intracellular Ki-67 in Mer^+^ KCs after 1 week of UNC2250 (red histogram). **(L)** Quantification of Csf1r MFI (left) and flow cytometry analysis (right) in F4/80^hi^ KCs after 1 week of UNC2250 treatment. Data represent at least 2 independent experiments with 3 to 5 mice per group. Bar graphs represent means +/- SEM. * *p* < 0.05.

Given their expression of Csf1r and Mer, we hypothesized that tissue-resident KCs would be significantly affected by inhibition of these receptors in vivo. PLX5622 is a small molecule that inhibits the Csf1r tyrosine kinase with an IC_50_ of <10 nM.[19] PLX5622 treatments were given for 1, 2, and 4 weeks. Treatments increased liver weights in quiescent livers and therefore, baseline liver-to-body weight ratios were increased (**S3A Fig**). Administration of PLX5622 for 2 weeks resulted in a 50% reduction in the percentage of F4/80^hi^ KCs among CD45^+^ NPCs, while the percentage of CD11b^hi^ KCs was unaffected (**Fig 2A and 2B**). PLX5622 resulted in minor alterations in percentages of other NPCs, mainly a mild increase in lymphocytes (**S2A Fig**). Administration of AFS98, an anti-Csf1r antibody, produced similar results, reducing the percentage of F4/80^hi^ KCs by half, while preserving CD11b^hi^ KCs (**Fig 2C and 2D**). PLX5622 administration also markedly decreased intracellular Ki-67 in F4/80^hi^ KCs, but not in CD11b^hi^ KCs (**Fig 2E**). As expected, PLX5622 selectively reduced the Mer^+^Csf1r^+^ subset of F4/80^hi^ KCs, by approximately 50% (**Fig 2B and 2F**). Overall, these data show that half of tissue-resident Mer^+^ KCs express and rely on Csf1r.

Clodronate liposomes are commonly used to deplete macrophages,[29] so we sought to investigate their effects on the F4/80^hi^ KC subsets we had identified. As expected, intravenous administration of clodronate liposomes nearly eradicated F4/80^hi^ KCs, consistent with their phagocytic function (**Fig 2G**). There was a small, residual population of Mer^+^Csf1r^-^ KCs (**Fig 2H** and **S2B Fig**). Clodronate liposomes also decreased Ki-67 on F4/80^hi^ KCs (**S2C Fig**) and increased Ly6c^hi^ inflammatory monocytes, as well as neutrophils and plasmacytoid dendritic cells (**Fig 2G**). Notably, control PBS liposomes reduced F4/80^hi^ KCs by about 25% while increasing the relative proportion of inflammatory CD11b^hi^ KCs and Ly6c^hi^ monocytes (**Fig 2G**). Taken together, clodronate liposomes do not simply deplete F4/80^hi^ KCs, as many other liver immune cells are affected, and control liposomes also have confounding effects.

Next, we examined the biological effects of inhibiting Mer. We administered UNC2250, a small molecule inhibitor selective for Mer with an IC_50_ of 1.7 nM and 160- and 60-fold relative selectivity compared to the related TAM kinases Axl and Tyro-3, respectively.[20] UNC2250 decreased phosphor-Mer in whole-liver lysates (**Fig 2I**). At different dosing schedules and amounts, UNC2250 did not deplete F4/80^hi^ or CD11b^hi^ KCs subsets (**Fig 2J**), or reduce Ki-67 staining of Mer^+^ KCs (**Fig 2K**). Furthermore, macrophage polarization and activation markers were not affected **S2D Fig**). Mer inhibition also did not affect Csf1r expression in F4/80^hi^ KCs (**Fig 2L**). The only appreciable change with UNC2250 administration was a compensatory increase in expression of the tyrosine kinase Kit in Mer^+^ KCs (**S2E Fig**). These data indicate that Mer inhibition does not impact the survival of KCs.

### Csf1r inhibition delays liver regeneration

**Fig 3:**
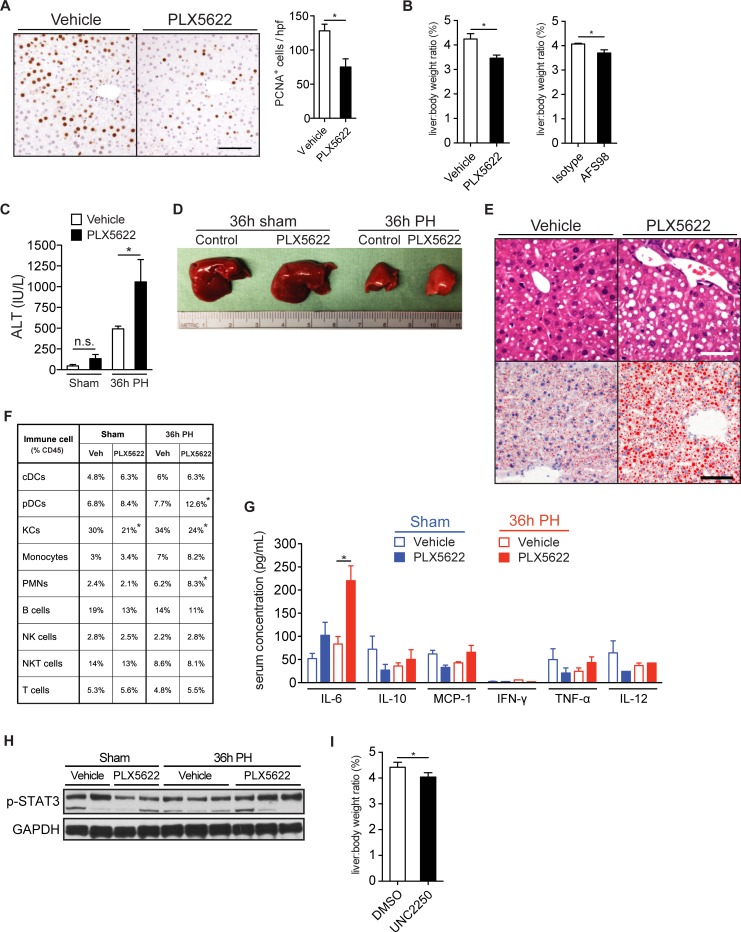
Csf1r or Mer inhibition delays liver regeneration. **(A)** Photomicrographs showing immunohistochemistry for PCNA in mouse livers (left) and quantification of PCNA^+^ cells counted per high power field (hpf) (right) 36h after PH. Scale bar, 100 μm. **(B)** Liver-to-body weight ratio (LBWR) 7d after PH in mice receiving PLX5622 (left) or AFS98 (right). **(C)** Serum ALT 36h after PH in mice receiving PLX5622. **(D)** Macroscopic photograph of livers from mice receiving PLX5622 36h after PH or sham surgery. **(E)** Photomicrographs of mouse livers 36h after PH stained with hematoxylin and eosin (top) or Oil red O (bottom). Scale bars, 100 μm. **(F)** Liver NPCs as a percentage of CD45^+^ cells 36h after PH or sham surgery. *p < 0.05 by ANOVA. **(G)** Phospho-STAT3 protein expression in whole-liver lysates by Western blot after a 36h PH or sham surgery in mice treated with PLX5622 for 1 week before the surgery. **(H)** Serum cytokine levels 36h after PH or sham surgery as measured by cytometric bead array. (**I)** LBWR 7d after PH in mice receiving UNC2250. Data represent at least 2 independent experiments with 3 to 5 mice per group. Bar graphs represent means +/- SEM. *p < 0.05.

KC depletion by clodronate liposomes is known to delay LR.[8–10] We investigated whether suppression of the specific F4/80^hi^ KC subsets was sufficient to delay LR. We administered PLX5622 to mice and then performed a two-thirds PH. PLX5622 significantly decreased the amount of PCNA^+^ regenerating cells at 36h after PH (**Fig 3A**). More importantly, PLX5622 and AFS98 each significantly delayed LR, as indicated by a reduction in liver-to-body weight ratios of 20% and 10%, respectively, at 7 days after PH (**Fig 3B**). PLX5622 also increased liver injury at 36h after PH, with elevated serum ALT (**Fig 3C**) and increased macroscopic (**Fig 3D**) and microscopic (**Fig 3E**) hepatic steatosis. AFS98 antibody had very similar effects at 36h and elevated serum ALT at 7d (**S3B and S3C Fig and Fig 3D**). Serum ALT remained elevated in PLX5622-treated mice up to 7 days after PH, and this effect was not due to Csf1r inhibition alone, since it was absent in the PLX5622-treated mice that underwent sham surgery (**S3E Fig).** In animals treated with PLX5622, sham operation did not induce significant changes in numbers of other NPCs besides KCs, while PH increased plasmacytoid dendritic cells and neutrophils (**Fig 3F**). IL-6 is a critical cytokine for normal LR,[30, 31] yet excessive IL-6 has been shown to be detrimental.[32] Even though PLX5622-treated mice had higher serum levels of IL-6 (**Fig 3G**) and higher whole-liver phospho-STAT3 (downstream from IL-6 receptor; **Fig 3H**) at 36h after PH, LR was delayed. Meanwhile, the other serum cytokines that were tested were not affected.

Finally, mice given UNC2250 had just modestly delayed LR, as shown by a 10% reduction in liver-to-body weight ratio (**Fig 3I**). At 36h after PH, there were no significant differences in PCNA^+^ regenerating cells, serum ALT, circulating serum cytokines, or NPC composition in mice treated with UNC2250 that underwent LR. In contrast, Mer^-/-^ mice did not have delayed LR with equal liver-to-body weight ratios to control Mer^+/+^ at 7 days (liver-to-body weight ratio means; 3.9 vs. 4.08, respectively; p = 0.40). Collectively, these data suggest that LR is sufficiently delayed by inhibiting KCs expressing Mer or Csf1r.

### Csf1r or Mer inhibition diminishes KC cytokine production in vitro

**Fig 4:**
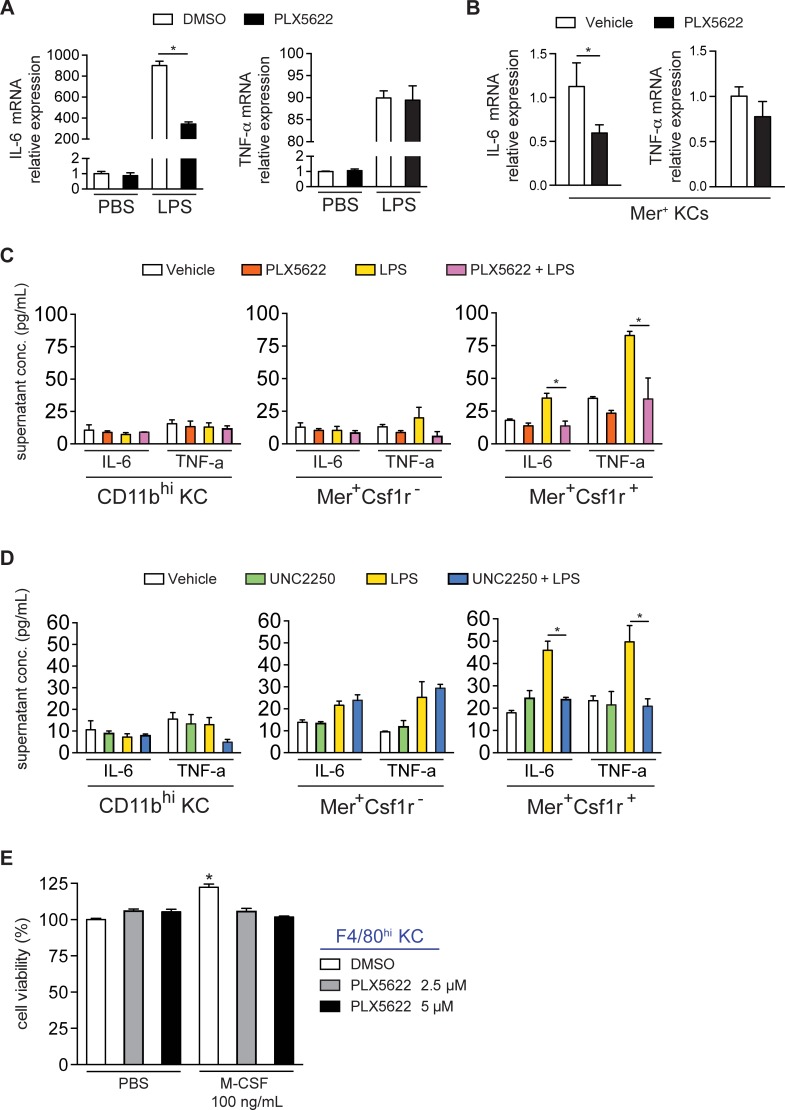
Csf1r or Mer inhibition diminishes KC cytokine production in vitro. **(A)** IL-6 (left) and TNF-α (right) as measured by quantitative PCR in freshly isolated F4/80^hi^ KCs cultured overnight with PLX5622 and then treated with LPS for 4h. **(B)** IL-6 (left) and TNF-α (right) as measured by quantitative PCR in freshly isolated Mer^+^ KCs from wild-type mice treated in vivo with 1 week of PLX5622. **(C, D)** Supernatant IL-6 and TNF-α levels measured by cytometric bead array in sorted and cultured CD11b^hi^, Mer^+^Csf1r^-^, and Mer^+^Csf1r^+^ KCs after overnight with either **(C)** PLX5622 or **(D)** UNC2250 and then stimulated with LPS for 4h. **(E)** Viability of F4/80^hi^ KCs freshly isolated and cultured for 72h with PLX5622 and M-CSF. Data represent at least 2 independent experiments with 3 to 5 mice per group. Bar graphs represent means +/- SEM and * *p* < 0.05.

Cytokine production from the liver inflammatory milieu plays a paramount role in LR, with IL-6 and TNF-α having particular importance.[30, 31] Therefore, we investigated the cytokine production of the KC subsets in vitro. When F4/80^hi^ KCs were pretreated with PLX5622, messenger RNA for IL-6, but not for TNF-α, was decreased in response to LPS (**Fig 4A**). Similar findings were observed in isolated Mer^+^ KCs mRNA from mice treated with PLX5622 in vivo (**Fig 4B**). To assess cytokine production more specifically, we sorted CD11b^hi^, CD11b^int^Mer^+^Csf1r^-^, and CD11b^int^Mer^+^Csf1r^+^ KCs. After stimulation with LPS, Mer^+^Csf1r^+^ KCs produced the most IL-6 and TNF-α, and this response was inhibited by PLX5622 (**Fig 4C**) and UNC2250 (**Fig 4D**). Notably, PLX5622 did not induce cell death of F4/80^hi^ KCs in vitro. However, PLX5622 blocked the M-CSF-mediated increase in F4/80^hi^ KC viability (**Fig 4E**).

## Discussion

KCs are located in the liver sinusoids where they detect pathogens and foreign antigens in the portal vein and play a central role in innate immunity.[1, 33] Initially, tissue-resident macrophages were thought to originate from circulating mononuclear cells that arise from the bone marrow and migrate to the periphery. Subsequent data have now demonstrated that most tissue macrophages arise from embryonic progenitors and are long-lived and self-renewing.[26, 27, 28, 34, 35] Two principal KC subsets have been described based on F4/80 and CD11b expression.[3, 4, 6] CD32 and CD68 have also been utilized to characterize KCs.[3, 5] However, CD32 is expressed on other cells, such as B cells. Meanwhile, CD68 stains both CD11b^hi^ and F4/80^hi^ KCs (**Fig 1B**), and is also present at low levels on some dendritic cells. It is known that CD68 is also detectable in lymphocytes, fibroblasts, and endothelial cells.[36] Furthermore, intracellular staining for CD68 requires cell fixation, thereby preventing subsequent functional analysis in vitro. Here, we sought to investigate other markers of KCs and showed that nearly all F4/80^hi^ KCs were Mer^+^. Notably, eosinophils expressed intermediate amounts of F4/80 and therefore may contaminate F4/80^+^ NPCs if the eosinophil marker Siglec-F is not used. Mer was not expressed by other liver NPCs, so it appears to be a more specific marker of KCs than F4/80 and CD68. Taken together, we define liver tissue-resident macrophages as CD45^+^Mer^+^.

F4/80^hi^ KCs are known to depend on the growth factor receptor Csf1r. Unexpectedly, though, only half of Mer^+^ KCs expressed Csf1r. To study Csf1r expression by KCs, we used a Csf1r-GFP transgenic mouse[17] because our collagenase-based liver NPC isolation protocol degraded Csf1r, making it undetectable by conventional flow cytometry. Mer^+^Csf1r^+^ and Mer^+^Csf1r^-^ KCs had distinct expression of a panel of macrophage markers and divided at different rates based on intracellular Ki-67 staining. Further investigation is necessary to determine whether these two subsets represent different developmental or maturational stages of KCs.

To study the function of individual KC subsets in vivo, it would be ideal to deplete them without significantly altering other liver NPCs. While clodronate liposomes have been used widely to deplete F4/80^hi^ KCs, it is well recognized that both clodronate and PBS liposomes have confounding effects on NPCs,[29] as we further documented here. In contrast, we found that Csf1r inhibition specifically targeted the Csf1r^+^ subset of F4/80^hi^Mer^+^ KCs, but had only minor effects on other liver NPCs. Notably, compensatory mechanisms appeared to develop after four weeks of Csf1r inhibition by PLX5622 treatment, since the percentage of F4/80^hi^ cells in the liver increased (**Fig 2A**) and Csf1r^+^ cells were especially apparent by immunohistochemistry (**S3F Fig**). Furthermore, while one week of PLX5622 treatment reduced blood Ly6c^hi^CCR2^+^ inflammatory monocytes by 25%, there was a monocyte surge above baseline at 2 weeks, and the percentage of blood monocytes normalized by 4 weeks of treatment (**S3G Fig**). Finally, after 1 week of Csf1r inhibition, expression of the growth factor receptor Flt-3 in liver and spleen Ly6c^hi^CCR2^+^ inflammatory monocytes increased (**S3H Fig**). In contrast to Csf1r inhibition, Mer inhibition, at least with the compound we tested, did not affect the percentage or proliferation of Mer^+^ KCs. In fact, Mer^-/-^ mice had a normal percentage of F4/80^+^ KCs and normal LR. Since there may be compensatory mechanisms in this knockout mouse, we did not perform further experiments in them. However, given that the Mer inhibitor delayed regeneration raises the possibility of an off target effect of the inhibitor. Thus, Csf1r appears to be more important than Mer in the viability of KCs. Nevertheless, Mer inhibition did increase the expression of Kit in Mer^+^ KCs, indicating that Mer contributes to KC homeostasis and Kit may compensate for its function.

Recently, a group constructed a mouse designed to allow depletion of F4/80^hi^ KCs; the mouse has a diphtheria-toxin receptor gene (DTR) driven by the C-type lectin Clec4f promoter, which is active specifically in liver macrophages.[23] F4/80^hi^ KCs were depleted after a single dose of diphtheria toxin, although the effect on other liver NPCs was not detailed. In our lab, we investigated KC depletion in two other DTR models. Liver CD11b^hi^ KCs were not depleted in CD11b-DTR mice[37] despite different diphtheria toxin doses and schedules, even though other CD11b^hi^ macrophages were depleted, including CD11b^hi^ peritoneal macrophages. In contrast, diphtheria toxin depleted both KC subsets in CCR2-DTR mice[38], as well as conventional and plasmacytoid dendritic cells. Another limitation of using CD11b-DTR or CCR2-DTR mice, or even Csf1r inhibition, is that macrophages outside of the liver are also affected.

Activation of the innate immune system is a hallmark of LR. The role of F4/80^hi^ KCs in LR has been well documented through the use of clodronate liposomes.[8–10] Another group described impaired LR in the osteopetrotic mouse (M-CSF knockout),[39] underscoring the importance of mature body macrophages during LR. The role of Csf1r signaling in developmentally normal mice during LR has not been documented previously. We found that disruption of Mer^+^Csf1r^+^ KCs via Csf1r inhibition is sufficient to increase liver injury and impair LR, as shown by lower liver-to-body weight ratios. To study whether Csf1r stimulation could enhance LR in wild-type mice, we administered M-CSF before and after PH. Despite expansion of Mer^+^ KCs in vivo by as much as 20%, liver-to-body weight ratios were equivalent at 4 and 7 days after PH (**S3I Fig**). Csf1r also appeared to be important to baseline liver homeostasis in the absence of PH, since Csf1r inhibition alone increased liver-to-body weight ratios after 1, 2, and 4 weeks of treatment (**S3A Fig**).

Csf1r inhibition reduced IL-6 and TNF-α production in vitro by Mer^+^Csf1r^+^ KCs, but not Mer^+^Csf1r^-^ KCs. Meanwhile, disruption of Mer signaling with a potent and selective Mer inhibitor also delayed LR, even though the percentage and proliferation of Mer^+^ KCs remained unchanged. However, Mer inhibition also reduced the production of IL-6 and TNF-α by Mer^+^Csf1r^+^ KCs in vitro. These data are consistent with the importance of local cytokine production by Mer^+^ KCs during hepatocyte priming in the initial phases of LR. Local production of IL-6 is known to be critical for the priming phase of LR.[40] While STAT3 activation is essential, [30, 31] excessive IL-6 can actually be detrimental.[36] Other groups showed a failure to mount an adequate circulating cytokine response after LR in animals treated with clodronate liposomes.[8–10] In contrast, we showed that despite higher levels of circulating IL-6 during the priming phase, Csf1r inhibition still delayed LR, consistent with the importance of local production of IL-6 and TNF-α within the regenerating liver.

Thus, tissue-resident macrophages of the liver are more specifically defined by Mer expression than by F4/80 expression. Two subsets of Mer^+^ KCs are defined by Csf1r expression. Csf1r inhibition selectively decreased the percentage, proliferation, and functioning of Mer^+^Csf1r^+^ KCs, and was sufficient to alter baseline liver homeostasis and delay LR. Mer inhibition also affected LR, without affecting the percentage or proliferation of Mer^+^ KCs. Csf1r or Mer inhibition specifically reduced cytokine expression of Mer^+^Csf1r^+^ KCs in vitro. Csf1r and Mer signaling pathways are appealing for further study in LR and other models of liver injury.

## Supporting information

S1 Fig **(A)** Flow cytometry gating strategy of B6 liver NPCs. Eosinophils (CD11b^hi^Siglec-F^+^) expressed intermediate amounts of F4/80 and were excluded. **(B)** Flow cytometry histograms of TAM family receptor expression in liver NPCs. **(C)** Immunofluorescence photomicrographs of mouse livers stained for F4/80, Csf1r, and nuclear DAPI. White asterisks indicate autofluorescent red blood cells in the liver sinusoids. Scale bars top 100 μm, bottom 20 μm. **(D)** TAM family tyrosine kinase receptor expression in Mer^+^Csf1r^-^ and Mer^+^Csf1r^+^ KCs by flow cytometry. Data represent at least 2 independent experiments with 3 to 5 mice per group.
(EPS)

S2 Fig **(A)** Changes in liver NPCs with PLX5622 treatment. *p < 0.05 by Student’s t-test. **(B)** Expression of Mer (left) and Csf1r (right) on KCs after administration of clodronate liposomes to Csf1r-GFP mice. **(C)** Intracellular Ki-67 in F4/80^hi^ KC treated with PBS, PBS liposomes (PBS lip) or clodronate liposomes (CLO lip). **(D)** Expression of MHC II, CD11c, and CD86 in F4/80^hi^ KCs from treated with UNC2250 for 1 week. **(E)** Quantification of Kit expression in Mer^+^Csf1r^+^ and Mer^+^Csf1r^-^ KCs and representative Kit staining in F4/80hi KCs (right) from mice treated with UNC2250 for 1 week. Data represent at least 2 independent experiments with 3 to 5 mice per group. Bar graphs represent means +/- SEM.
(EPS)

S3 Fig **(A)** Changes in liver-to-body weight ratios in quiescent livers after 1, 2 and 4 weeks of Csf1r inhibition with PLX5622. Blue squares: 16-week old B6 male mice; red circles: 10-week old B6 male mice; brown squares: 9-week old B6 male mice; orange inverted triangles: 12-week old B6 male mice. **(B)** Photomicrographs showing immunohistochemistry for PCNA in mouse livers 36h after PH. AFS98: anti-Csf1r antibody. Black scale bar, 100 μm. **(C)** Photomicrographs of mouse livers 36h after PH stained with hematoxylin and eosin. AFS98: anti-Csf1r antibody. Black scale bar, 100 μm. **(D)** Serum ALT 7d after PH in mice receiving the anti-Csf1r antibody, AFS98. **(E)** Serum ALT 7 days after PH and sham surgery. **(F)** F4/80, Csf1r, and nuclear DAPI immunofluorescence photomicrographs of mouse livers treated for 4 weeks with Csf1r inhibition, showing recovery of Kupffer cells. White scale bars, 100 μm. **(G)** Flow cytometry showing the effects of 1, 2, and 4 weeks of PLX5622 treatment on blood Ly6c^hi^CCR2^+^ inflammatory monocytes. Black arrow and unfilled area: PMN (neutrophils); pink filled area: Ly6c^hi^CCR2^+^ inflammatory monocytes. **(H)** Flow cytometry expression of the growth factor receptor Flt-3 in liver and spleen Ly6c^hi^CCR2^+^ inflammatory monocytes after 1 week of PLX5622 treatments. **(I)** Flow cytometry showing expansion of quiescent B6 liver Mer^+^ KCs with administration of exogenous M-CSF (left); equivalent liver-to-body weight ratios 4 (middle) and 7 (right) days after PH. All graphs represent means +/- SEM. *p < 0.05. n.s.: not significant.
(PDF)

## Abbreviations

KCs: Kupffer cells
Csf1r: colony-stimulating factor 1 receptor
LR: liver regeneration
PH: partial hepatectomy
ALT: alanine aminotransferase
PCNA: proliferating cell nuclear antigen
NPCs: liver nonparenchymal cells
SEM: standard error of the mean
DTR: diphtheria-toxin receptor gene

## References

[1] Dixon LJ, Barnes M, Tang H, Pritchard MT, Nagy LE. Kupffer cells in the liver. Compr Physiol. 2013;3(2):785–97. 10.1002/cphy.c120026 . 23720329 PMC4748178

[2] Holt MP, Cheng L, Ju C. Identification and characterization of infiltrating macrophages in acetaminophen-induced liver injury. J Leukoc Biol. 2008;84(6):1410–21. 10.1189/jlb.0308173 . 18713872 PMC2614594

[3] Kinoshita M, Uchida T, Sato A, Nakashima M, Nakashima H, Shono S, et al Characterization of two F4/80-positive Kupffer cell subsets by their function and phenotype in mice. J Hepatol. 2010;53(5):903–10. 10.1016/j.jhep.2010.04.037 . 20739085

[4] Ramachandran P, Pellicoro A, Vernon MA, Boulter L, Aucott RL, Ali A, et al Differential Ly-6C expression identifies the recruited macrophage phenotype, which orchestrates the regression of murine liver fibrosis. Proc Natl Acad Sci U S A. 2012;109(46):E3186–95. 10.1073/pnas.1119964109 . 23100531 PMC3503234

[5] Ikarashi M, Nakashima H, Kinoshita M, Sato A, Nakashima M, Miyazaki H, et al Distinct development and functions of resident and recruited liver Kupffer cells/macrophages. J Leukoc Biol. 2013;94(6):1325–36. 10.1189/jlb.0313144 . 23964119

[6] Klein I, Cornejo JC, Polakos NK, John B, Wuensch SA, Topham DJ, et al Kupffer cell heterogeneity: functional properties of bone marrow derived and sessile hepatic macrophages. Blood. 2007;110(12):4077–85. 10.1182/blood-2007-02-073841 . 17690256 PMC2190614

[7] Michalopoulos GK. Principles of liver regeneration and growth homeostasis. Compr Physiol. 2013;3(1):485–513. 10.1002/cphy.c120014 . 23720294

[8] Abshagen K, Eipel C, Kalff JC, Menger MD, Vollmar B. Loss of NF-kappaB activation in Kupffer cell-depleted mice impairs liver regeneration after partial hepatectomy. Am J Physiol Gastrointest Liver Physiol. 2007;292(6):G1570–7. 10.1152/ajpgi.00399.2006 . 17322066

[9] Meijer C, Wiezer MJ, Diehl AM, Schouten HJ, Schouten HJ, Meijer S, et al Kupffer cell depletion by CI2MDP-liposomes alters hepatic cytokine expression and delays liver regeneration after partial hepatectomy. Liver. 2000;20(1):66–77. . 10726963 10.1034/j.1600-0676.2000.020001066.x

[10] Selzner N, Selzner M, Odermatt B, Tian Y, Van Rooijen N, Clavien PA. ICAM-1 triggers liver regeneration through leukocyte recruitment and Kupffer cell-dependent release of TNF-alpha/IL-6 in mice. Gastroenterology. 2003;124(3):692–700. 10.1053/gast.2003.50098 . 12612908

[11] Hume DA, MacDonald KP. Therapeutic applications of macrophage colony-stimulating factor-1 (CSF-1) and antagonists of CSF-1 receptor (CSF-1R) signaling. Blood. 2012;119(8):1810–20. 10.1182/blood-2011-09-379214 . 22186992

[12] Yu W, Chen J, Xiong Y, Pixley FJ, Yeung YG, Stanley ER. Macrophage proliferation is regulated through CSF-1 receptor tyrosines 544, 559, and 807. J Biol Chem. 2012;287(17):13694–704. 10.1074/jbc.M112.355610 . 22375015 PMC3340183

[13] Lemke G. Biology of the TAM receptors. Cold Spring Harb Perspect Biol. 2013;5(11):a009076 10.1101/cshperspect.a009076 . 24186067 PMC3809585

[14] Rothlin CV, Carrera-Silva EA, Bosurgi L, Ghosh S. TAM receptor signaling in immune homeostasis. Annu Rev Immunol. 2015;33:355–91. 10.1146/annurev-immunol-032414-112103 . 25594431 PMC4491918

[15] Rothlin CV, Ghosh S, Zuniga EI, Oldstone MB, Lemke G. TAM receptors are pleiotropic inhibitors of the innate immune response. Cell. 2007;131(6):1124–36. 10.1016/j.cell.2007.10.034 . 18083102

[16] Gautier EL, Shay T, Miller J, Greter M, Jakubzick C, Ivanov S, et al Gene-expression profiles and transcriptional regulatory pathways that underlie the identity and diversity of mouse tissue macrophages. Nat Immunol. 2012;13(11):1118–28. 10.1038/ni.2419 . 23023392 PMC3558276

[17] Burnett SH, Kershen EJ, Zhang J, Zeng L, Straley SC, Kaplan AM, et al Conditional macrophage ablation in transgenic mice expressing a Fas-based suicide gene. J Leukoc Biol. 2004;75(4):612–23. 10.1189/jlb.0903442 . 14726498

[18] Lu Q, Gore M, Zhang Q, Camenisch T, Boast S, Casagranda F, et al Tyro-3 family receptors are essential regulators of mammalian spermatogenesis. Nature. 1999;398(6729):723–8. 10.1038/19554 . 10227296

[19] Coniglio SJ, Eugenin E, Dobrenis K, Stanley ER, West BL, Symons MH, et al Microglial stimulation of glioblastoma invasion involves epidermal growth factor receptor (EGFR) and colony stimulating factor 1 receptor (CSF-1R) signaling. Mol Med. 2012;18:519–27. 10.2119/molmed.2011.00217 . 22294205 PMC3356419

[20] Zhang W, Zhang D, Stashko MA, DeRyckere D, Hunter D, Kireev D, et al Pseudo-cyclization through intramolecular hydrogen bond enables discovery of pyridine substituted pyrimidines as new Mer kinase inhibitors. J Med Chem. 2013;56(23):9683–92. 10.1021/jm401387j . 24195762 PMC3980660

[21] Higgins GM AR. Experimental pathology of liver. I. Restoration of liver of white rat following partial surgical removal. Arch Pathol. 1930;12:186–202.

[22] Pillarisetty VG, Shah AB, Miller G, Bleier JI, DeMatteo RP. Liver dendritic cells are less immunogenic than spleen dendritic cells because of differences in subtype composition. J Immunol. 2004;172(2):1009–17. . 14707074 10.4049/jimmunol.172.2.1009

[23] Scott CL, Zheng F, De Baetselier P, Martens L, Saeys Y, De Prijck S, et al Bone marrow-derived monocytes give rise to self-renewing and fully differentiated Kupffer cells. Nat Commun. 2016;7:10321 10.1038/ncomms10321 . 26813785 PMC4737801

[24] Amiya T, Nakamoto N, Chu PS, Teratani T, Nakajima H, Fukuchi Y, et al Bone marrow-derived macrophages distinct from tissue-resident macrophages play a pivotal role in Concanavalin A-induced murine liver injury via CCR9 axis. Sci Rep. 2016;6:35146 10.1038/srep35146 . 27725760 PMC5057133

[25] Zagorska A, Traves PG, Lew ED, Dransfield I, Lemke G. Diversification of TAM receptor tyrosine kinase function. Nat Immunol. 2014;10:920–8. 10.1038/ni.2986 25194421 PMC4169336

[26] Schulz C, Gomez Perdiguero E, Chorro L, Szabo-Rogers H, Cagnard N, Kierdorf K, et al A lineage of myeloid cells independent of Myb and hematopoietic stem cells. Science. 2012;336(6077):86–90. 10.1126/science.1219179 . 22442384

[27] Jenkins SJ, Ruckerl D, Cook PC, Jones LH, Finkelman FD, van Rooijen N, et al Local macrophage proliferation, rather than recruitment from the blood, is a signature of TH2 inflammation. Science. 2011;332(6035):1284–8. 10.1126/science.1204351 21566158 PMC3128495

[28] Jenkins SJ, Ruckerl D, Thomas GD, Hewitson JP, Duncan S, Brombacher F, et al IL-4 directly signals tissue-resident macrophages to proliferate beyond homeostatic levels controlled by CSF-1. J Exp Med. 2013;210(11):2477–91. 10.1084/jem.20121999 24101381 PMC3804948

[29] Van Rooijen N, Sanders A. Liposome mediated depletion of macrophages: mechanism of action, preparation of liposomes and applications. J Immunol Methods. 1994;174(1–2):83–93. . 8083541 10.1016/0022-1759(94)90012-4

[30] Cressman DE, Diamond RH, Taub R. Rapid activation of the Stat3 transcription complex in liver regeneration. Hepatology. 1995;21(5):1443–9. . 7737651

[31] Cressman DE, Greenbaum LE, DeAngelis RA, Ciliberto G, Furth EE, Poli V, et al Liver failure and defective hepatocyte regeneration in interleukin-6-deficient mice. Science. 1996;274(5291):1379–83. . 8910279 10.1126/science.274.5291.1379

[32] Wustefeld T, Rakemann T, Kubicka S, Manns MP, Trautwein C. Hyperstimulation with interleukin 6 inhibits cell cycle progression after hepatectomy in mice. Hepatology. 2000;32(3):514–22. 10.1053/jhep.2000.16604 . 10960443

[33] Kolios G, Valatas V, Kouroumalis E. Role of Kupffer cells in the pathogenesis of liver disease. World J Gastroenterol. 2006;12(46):7413–20. 10.3748/wjg.v12.i46.7413 . 17167827 PMC4087584

[34] Gomez Perdiguero E, Geissmann F. Myb-independent macrophages: a family of cells that develops with their tissue of residence and is involved in its homeostasis. Cold Spring Harb Symp Quant Biol. 2013;78:91–100. 10.1101/sqb.2013.78.020032 . 24122769

[35] Hashimoto D, Chow A, Noizat C, Teo P, Beasley MB, Leboeuf M, et al Tissue-resident macrophages self-maintain locally throughout adult life with minimal contribution from circulating monocytes. Immunity. 2013;38(4):792–804. 10.1016/j.immuni.2013.04.004 . 23601688 PMC3853406

[36] Gottfried E, Kunz-Schughart LA, Weber A, Rehli M, Peuker A, Müller A, et al Expression of CD68 in non-myeloid cell types. Scand J Immunol. 2008 5;67(5):453–63. 10.1111/j.1365-3083.2008.02091.x . 18405323

[37] Duffield JS, Forbes SJ, Constandinou CM, Clay S, Partolina M, Vuthoori S, et al Selective depletion of macrophages reveals distinct, opposing roles during liver injury and repair. J Clin Invest. 2005;115(1):56–65. 10.1172/JCI22675 . 15630444 PMC539199

[38] Hohl TM, Rivera A, Lipuma L, Gallegos A, Shi C, Mack M, Pamer EG. Inflammatory monocytes facilitate adaptive CD4 T cell responses during respiratory fungal infection. Cell Host Microbe. 2009 11 19;6(5):470–81. 10.1016/j.chom.2009.10.007 . 19917501 PMC2785497

[39] Amemiya H, Kono H, Fujii H. Liver regeneration is impaired in macrophage colony stimulating factor deficient mice after partial hepatectomy: the role of M-CSF-induced macrophages. J Surg Res. 2011;165(1):59–67. 10.1016/j.jss.2009.08.008 . 20031174

[40] Aldeguer X, Debonera F, Shaked A, Krasinkas AM, Gelman AE, Que X, et al Interleukin-6 from intrahepatic cells of bone marrow origin is required for normal murine liver regeneration. Hepatology. 2002;35(1):40–8. 10.1053/jhep.2002.30081 . 11786958

