# Supplementary figures and images for: Csf1r or Mer inhibition delays liver regeneration via suppression of Kupffer cells
Source: PLoS One. 2019 May 1;14(5):e0216275. doi: 10.1371/journal.pone.0216275 (PMC6493758; doi:10.1371/journal.pone.0216275)

Supplemental Figure 3

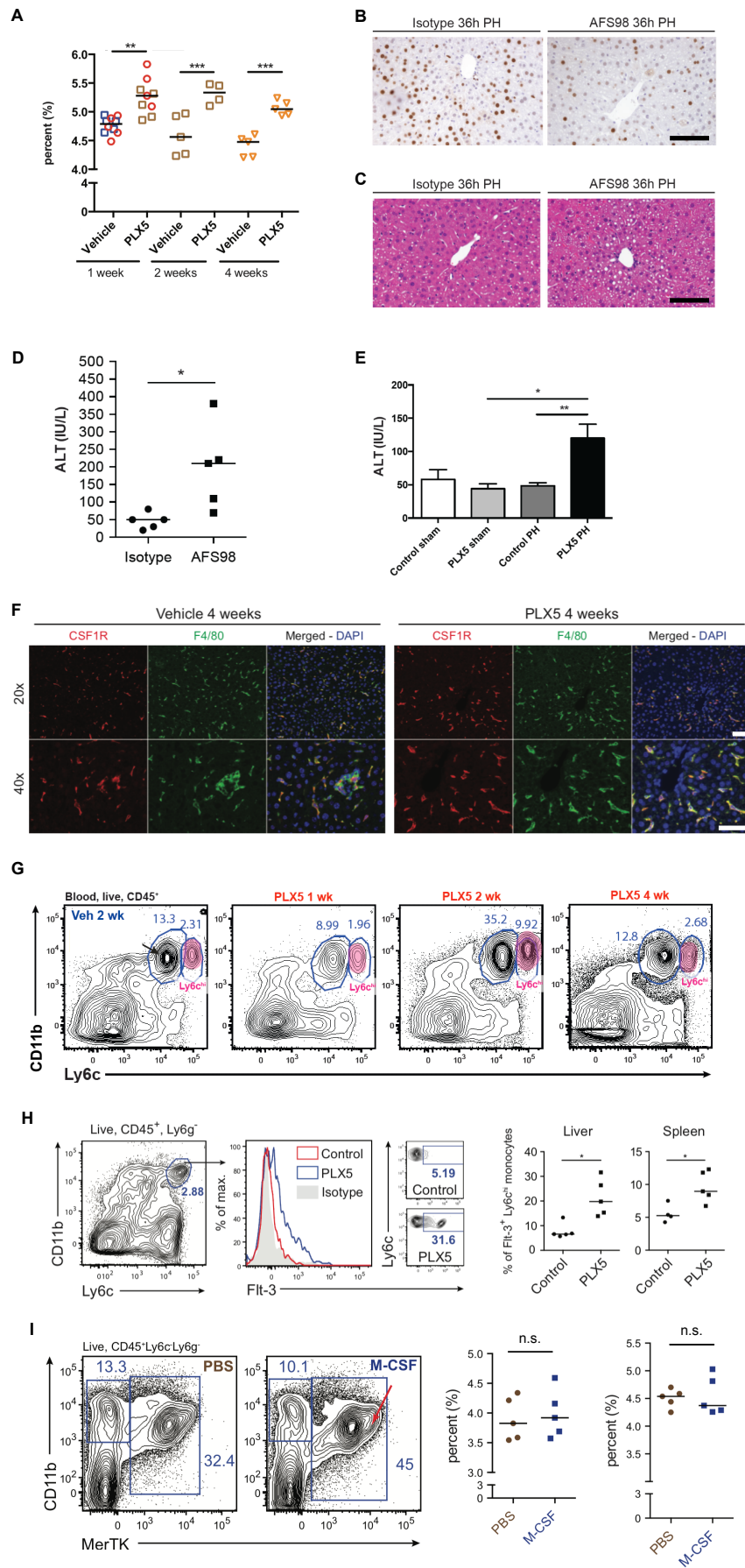

Supplement: S3 Fig — (A) Changes in liver-to-body weight ratios in quiescent livers after 1, 2 and 4 weeks of Csf1r inhibition with PLX5622. Blue squares: 16-week old B6 male mice; red circles: 10-week old B6 male mice; brown squares: 9-week old B6 male mice; orange inverted triangles: 12-week old B6 male mice. (B) Photomicrographs showing immunohistochemistry for PCNA in mouse livers 36h after PH. AFS98: anti-Csf1r antibody. Black scale bar, 100 μm. (C) Photomicrographs of mouse livers 36h after PH stained with hematoxylin and eosin. AFS98: anti-Csf1r antibody. Black scale bar, 100 μm. (D) Serum ALT 7d after PH in mice receiving the anti-Csf1r antibody, AFS98. (E) Serum ALT 7 days after PH and sham surgery. (F) F4/80, Csf1r, and nuclear DAPI immunofluorescence photomicrographs of mouse livers treated for 4 weeks with Csf1r inhibition, showing recovery of Kupffer cells. White scale bars, 100 μm. (G) Flow cytometry showing the effects of 1, 2, and 4 weeks of PLX5622 treatment on blood Ly6chiCCR2+ inflammatory monocytes. Black arrow and unfilled area: PMN (neutrophils); pink filled area: Ly6chiCCR2+ inflammatory monocytes. (H) Flow cytometry expression of the growth factor receptor Flt-3 in liver and spleen Ly6chiCCR2+ inflammatory monocytes after 1 week of PLX5622 treatments. (I) Flow cytometry showing expansion of quiescent B6 liver Mer+ KCs with administration of exogenous M-CSF (left); equivalent liver-to-body weight ratios 4 (middle) and 7 (right) days after PH. All graphs represent means +/- SEM. *p < 0.05. n.s.: not significant. (PDF) [file pone.0216275.s003.pdf]
